# Factors associated with non-carbapenemase mediated carbapenem resistance of Gram-negative bacteria: a retrospective case-control study

**DOI:** 10.1007/s10123-023-00405-6

**Published:** 2023-08-09

**Authors:** Marius Müller, Andrea Wiencierz, Christian Gehringer, Veronika Muigg, Stefano Bassetti, Martin Siegemund, Vladimira Hinic, Sarah Tschudin-Sutter, Adrian Egli

**Affiliations:** 1 Applied Microbiology Research, Department of Biomedicine, University of Basel, Basel, Switzerland; 2 Clinical Bacteriology and Mycology, University Hospital Basel, Basel, Switzerland; 3 Clinical Trial Unit, University Hospital Basel, Basel, Switzerland; 4 Internal Medicine, University Hospital Basel, Basel, Switzerland; 5 Intensive Care Medicine, Department of Acute Medicine, University Hospital Basel, Basel, Switzerland; 6 Department of Clinical Research, University of Basel, Basel, Switzerland; 7 Infectious Diseases and Hospital Epidemiology, University Hospital Basel, Basel, Switzerland; 8 Institute of Medical Microbiology, University of Zurich, Gloriastrasse 28/30, 8006 Zurich, Switzerland

**Keywords:** Carbapenem resistance, Gram-negative bacteria, Carbapenemase-negative, Pseudomonas aeruginosa

## Abstract

Infections with carbapenemase-producing Gram-negative bacteria are related to increased morbidity and mortality, yet little is known regarding infections caused by non-beta-lactamase mediated carbapenem-resistant bacteria. Our objective was to identify risk factors for, and the clinical impact of infections caused by carbapenem-resistant carbapenemase-negative *Enterobacterales* and *Pseudomonas aeruginosa*. This retrospective matched case-control study was performed at the University Hospital of Basel, Switzerland, in 2016. We focused on other resistance mechanisms by excluding laboratory-confirmed carbapenemase-positive cases. Carbapenem resistance was set as the primary endpoint, and important risk factors were investigated by conditional logistic regression. The clinical impact of carbapenem resistance was estimated using regression models containing the resistance indicator as explanatory factor and adjusting for potential confounders. Seventy-five cases of infections with carbapenem-resistant, carbapenemase-negative bacteria were identified and matched with 75 controls with carbapenem-susceptible infections. The matched data set was well-balanced regarding age, gender, and comorbidity. Duration of prior carbapenem treatment (OR 1.15, [1.01, 1.31]) correlated with resistance to carbapenems. Our study showed that patients with carbapenem-resistant bacteria stayed 1.59 times (CI [0.81, 3.14]) longer in an ICU. The analyzed dataset did not provide evidence for strong clinical implications of resistance to carbapenems or increased mortality. The duration of prior carbapenem treatment seems to be a strong risk factor for the development of carbapenem resistance. The higher risk for a longer ICU stay could be a consequence of a carbapenem resistance. In contrast to carbapenemase-producers, the clinical impact of carbapenamase-negative, carbapenem-resistant strains may be limited. Trial registration: The study design was prospectively approved by the local Ethics Commission on 10.08.2017 (EKNZ BASEC 2017-00222).

**Supplementary Information:**

The online version contains supplementary material available at 10.1007/s10123-023-00405-6.

## Introduction

Carbapenems are important beta–lactam antibiotics due to their broad spectrum of action. This allows the treatment against a wide range of antimicrobial resistant bacteria, including leading pathogens of hospital-acquired infections such as extended-spectrum beta-lactamase (ESBL)- (Paterson 2000) or AmpC-producing *Enterobacterales* (Metsini et al. 2018), and non-fermenting Gram-negative bacteria including *Pseudomonas aeruginosa* (Lautenbach et al. 2010). Carbapenem resistance has been associated with increased mortality rates, prolonged hospital stays, and higher hospitalization costs (Adams et al. 2020; Correa et al. 2013). Therefore, resistance against carbapenems is critical for treatment and patient management (Ferstl et al. 2017; Schwaber et al. 2008).

Several studies have examined risk factors for the development of carbapenem resistance. They suggest that the previous use of antibiotics and in particular of carbapenems prior to hospital stay, admission to the intensive care unit (ICU), required assistance in activities of daily living, and usage of central catheters favor the acquisition or selection of resistant strains (Correa et al. 2013; Jeon et al. 2008; Schwaber et al. 2008; Wang et al. 2016).

Four mechanisms of carbapenem resistance are recognized: production of carbapenem-hydrolyzing β-lactamases, expression of efflux pumps, mutations that influence penicillin-binding proteins (PBPs), and change of functional porins (Papp-Wallace et al. 2011). Most of the clinical studies focus on plasmid-acquired carbapenemases. Although it has been shown that deletions or single nucleotide polymorphisms cause changes in the aminoacid sequence and subsequent structure of the porin protein (Sanbongi et al. 2009), the clinical aspects of porin loss are not well investigated. The OprD porin protein of *P. aeruginosa* is strongly linked with carbapenem resistance (Sanbongi et al. 2009; Yoneyama and Nakae 1993). Additional studies further reported the importance of variations in porin expression in *Enterobacterales* (Doumith et al. 2009; Lavigne et al. 2013). Further mechanisms may evolve subsequently under the ongoing selective pressure induced by the broad use of carbapenems (Li et al. 2012).

The objective of this study was to identify the clinical determinants of carbapenemase-negative, carbapenem-resistant *Enterobacterales* as well as *P. aeruginosa*, and to explore the clinical implications of these resistances in a low-endemic setting of carbapenemases.

## Methods

### Design, setting, and ethics

The University Hospital Basel (USB) in Switzerland is a tertiary care hospital with a capacity of 855 beds and approximately 38,000 admissions per year. In Switzerland, carbapenemases in *Enterobacterales* are rare on a stable low level and constitute 142 isolates during 2016 in the whole country (Federal Office of Public Health and Federal Food Safety and Veterinary Office 2018). The study design was approved by the local Ethics Commission (EKNZ BASEC 2017-00222).

In this retrospective case-control study, we included as potential cases all hospitalized adult patients at the USB in 2016, who received antimicrobial susceptibility testing for *Enterobacterales or P. aeruginosa*. The *Enterobacterales* group included the following species: *Citrobacter freundii* group, *Enterobacter cloacae* group, *Klebsiella aerogenes*, *Escherichia coli*, *Serratia marcescens*, and *Klebsiella pneumoniae* group. Furthermore, species with resistances to the carbapenems ertapenem, imipinem, or meropenem were selected for further analysis. Due to weak activity of ertapenem against *P. aeruginosa (*Papp-Wallace et al. 2011*)*, the susceptibility testing of *P. aeruginosa* was performed on imipenem and meropenem. Cases with a pheno- or genotypically confirmed carbapenemase-positive isolate were excluded. Hospitalization below 24 h, insufficient documentation in the chart record, or multiple admissions for a single case were defined as additional exclusion criteria. In case of repeated bacterial isolates from the same patient, the first resistant isolate was taken.

To each of the carbapenem-resistant, carbapenemase-negative cases, a carbapenem-sensitive control was exactly matched according to the following criteria: bacterial species, ward type (medical, surgical, obstetrics and gynecology, and intensive care unit), and isolation source (blood, urine, respiratory material, and superficial swabs or deep tissue biopsies). The prioritized control was replaced with an alternative control in case of multiple selection, insufficient information, or hospitalization below 24 h.

In the primary analysis, the carbapenem resistance was set as the primary endpoint, and the most important risk factors were investigated. The clinical implications were examined in the secondary analysis regarding the endpoints: all-cause mortality during hospital stay (within 30 days after resistance test (RT)), total intensive care unit stay (days), hospital stay duration (days), rehospitalization at USB (yes/no), and duration of antibiotic treatment (days).

### Patient data collection

Data for all patients were gathered retrospectively by chart review. This included associated patient data separately for the time frame before and after the isolates were identified. The following information was collected: adapted Charlson comorbidity index (Charlson et al. 1994), transplantations, demographic information, and resistance profile of the isolate to other antibiotics. Previous intake of acetylsalicylic acid or antacids (at admission), previous surgical interventions (3 months prior to the RT), previous hospitalizations (1 month prior to the RT), and foreign body (in situ at the time of the RT) such as urinary catheter were recorded. The following data were gathered separately for the time frame before and after susceptibility testing as well as during the whole period: duration of antibiotic intake split into common antibiotic subgroups (1 month prior to the RT; RT till discharge), and duration of hospitalization and intensive care unit stay. Different variables like within-hospital mortality and rehospitalization at the USB (<1 month after discharge) completed the data base.

The data were recorded in an electronic case report form with the support of the freeware “Epidata” (version 4.2.0.0.) (EpiData Association 2017).

### Antibiotic susceptibility testing

**Fig. 1:**
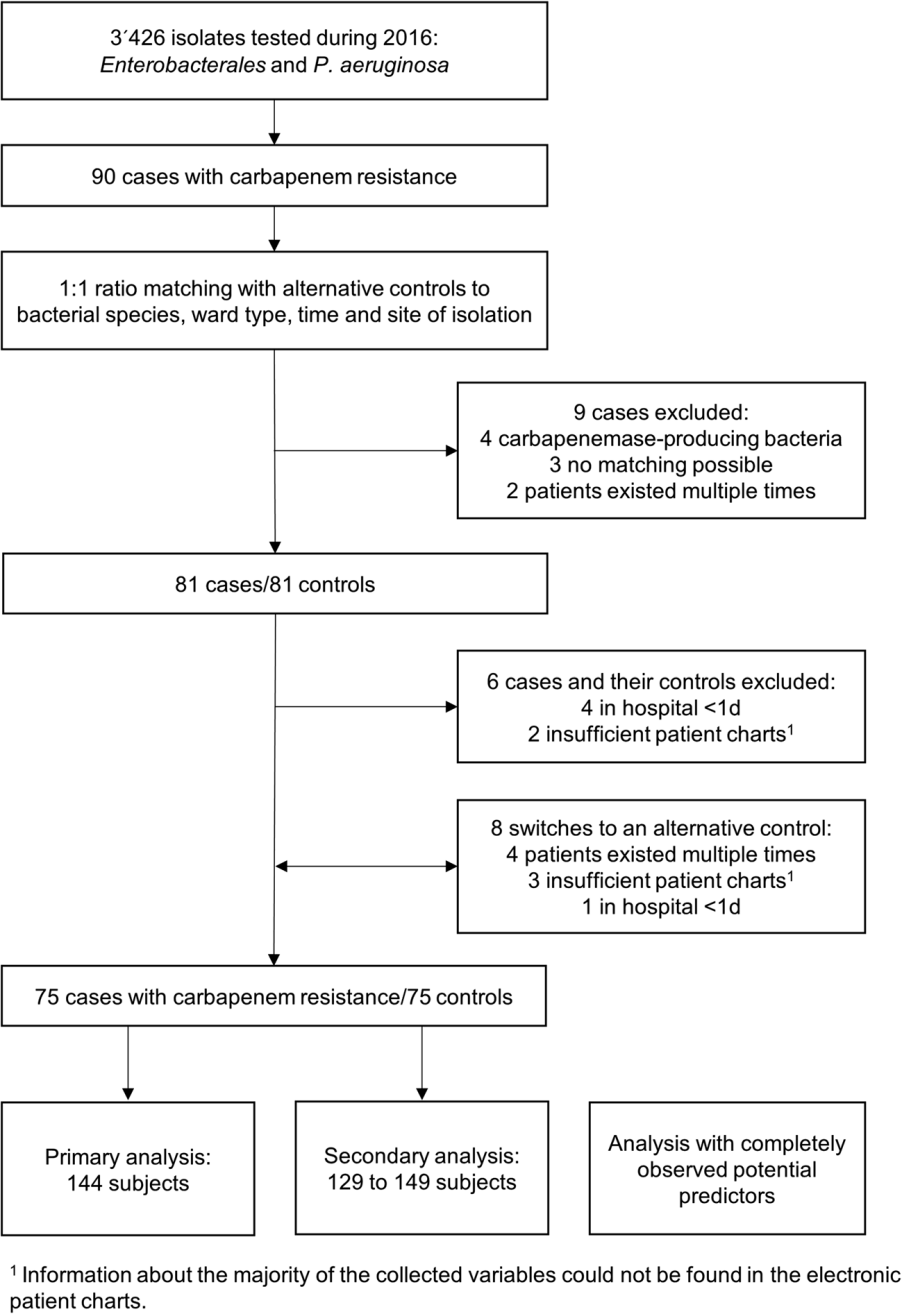
Flow diagram of the matching and exclusion process for the case-control study of carbapenem-resistant *Enterobacterales* and *P. aeruginosa* in Switzerland, 2016

We included all bacterial isolates found during routine diagnostics at the ISO-accredited diagnostic microbiology laboratory (Fig. 1). Antimicrobial susceptibility testing was performed according to EUCAST guidelines. Bacterial isolates were identified using MALDI-TOF mass spectrometry (Bruker Microflex, Bremen, Germany) with the mass-spectrum library and the MALDI Biotyper 3 software (OC 3.1, Bruker Daltonics) at standard conditions. Alternatively, we used the biochemical profile from VITEK2 (bioMérieux, Marcy-l’Étoile, France) for identification. Antimicrobial susceptibility profile was generated using the Gram-negative AST card (N242) on the VITEK 2 (bioMérieux) instrument. We used EUCAST recommendations for screening for carbapenemase production (Giske et al. 2013).

For screening purposes, we used specific selective chromogenic culture plates: CARBA-ID for carbapenemases (bioMérieux) and ESBL-ID for ESBL (bioMérieux) (Hinić et al. 2017). Identification of colonies growing on screening plates was performed using MALDI-TOF MS, followed by a phenotypic and/or genotypic confirmation of the corresponding resistance mechanism.

For phenotypic confirmation we used (i) the KPC, MBL, and OXA-48 confirm kit (ROSCO, Taastrup, Denmark) or (ii) for Metallo-Beta-Lactamases also a combined dual E-Test® MBL IP/IPI (bioMérieux) was used. These tests are based on a ratio interpretation by a MIC or inhibition zone, resulting from a combination of the carbapenem antibiotic with and without a carbapenemase inhibitor.

For genotypic confirmation, we used the following tests: (i) Xpert Carba-R (Cepheid, Sunnyvale, California) covering KPC, NDM, VIM, IMP-1, and OXA-48;(ii) eazyplex SuperBug CRE (Amplex, Gars am Inn, Germany) covering KPC, NDM, OXA-48, OXA-181, VIM, and the ESBL genes of the CTX-M-1 and CTX-M-9 group (Hinić et al. 2015) or whole genome sequencing.

### Statistics

#### Matching

Matching was done using R (version 3.3.0) and the MatchIt software package (Daniel et al. 2011; R Core Team 2016). Every carbapenem-resistant case was matched to five non-resistant controls by corresponding bacterial species, ward, and source of bacterial isolation (i.e., material category). Subsequently, a nearest neighbor analysis sorted the matched controls by the date the isolates were taken and prioritized controls that were closer to the date of the corresponding case. As the statistics was performed with one prioritized control per case, the remaining 4 controls were used as alternative options.

#### Outcomes and analysis

Prior to analysis, we determined an analytical plan following standard statistical procedures at the Clinical Trial Unit. The primary objective of this case-control study was to identify the determinants of non-carbapenemase mediated carbapenem resistance. For an overview, the baseline characteristics were summarized. Median, standardized mean difference (SMD), and standard deviation are shown unless otherwise indicated. When comparing two groups, the SMD measures the difference between the group means of a variable in terms of the average standard deviation of this variable within the two groups (Austin 2011).

Since we were dealing with an exactly matched data set, the primary analysis was based on conditional logistic regression, estimated using the R package survival (version 2.44-1.1) (Pearce 2016). Beforehand, the five potentially most important risk factors for a resistance were selected by the investigators: carbapenem treatment duration before RT (days), hospital stay duration before RT (days), ICU stay duration before RT (days), surgery before RT (yes/no), and Charlson comorbidity index (numeric score). To identify further relevant predictors among the baseline characteristics, we performed an automated variable selection based on Akaike’s information criterion (AIC) using the R package MASS (version 7.3-51.4). Using all patients with completely observed data, we performed a forward variable selection and a backward selection as sensitivity analysis. In addition, we conducted sensitivity analysis using two pragmatic imputation methods.

The average impact of carbapenem resistance on the secondary endpoints was estimated using regression models containing the resistance indicator as explanatory factor and controlling for further potential confounders. For the secondary endpoint death during hospital stay, we used logistic regression models, while the outcome total intensive care unit stay duration was analyzed with a hurdle model (Zeileis 2008). The results for hospital stay duration and duration of antibiotic treatment were obtained using (generalized) linear regression models. For each secondary outcome, we estimated one model controlling for a minimal set of potential confounders identified by the investigators and compared this with a model adjusting for a more comprehensive set of covariates.

## Results

During the study period 3,426 isolates of *Enterobacterales* and *P. aeruginosa* were tested for carbapenem susceptibility. Out of this cohort, 90 patients were identified with bacteria carrying one or more resistances to the carbapenems ertapenem, imipinem, or meropenem and selected for further analysis. Four of these 90 cases were tested positive for carbapenemases and subsequently excluded. Reasons for additional exclusions were hospitalization below 24 h, insufficient documentation in the chart record, or multiple admissions for a single case (Fig. 1). Eight controls were replaced due to their multiple selection or insufficient information.

We finally included retrospective data of 75 resistant cases matched with 75 controls sensitive to carbapenems. As the analysis could only be performed with completely observed variables, each analysis was performed with a different number of subjects. Depending on the selected potential predictors for each analysis, cases and controls with at least one incomplete variable had to be excluded. The variables were incomplete in case the information in the patient chart did not allow a statement. For example, as the information was taken from in hospital patient charts, no statement could be made about the mortality within 30 days where a patient left the hospital during this period without readmission.

Table S1 of the supplementary material shows the structure of the matching criteria. Forty-three (57.3%) of all 75 cases were tested positive to carbapenem-resistant *P. aeruginosa*. Moreover, respiratory material was the most common isolation site with 31/75 (41.3%) cases and 30/75 (40%) isolates were detected in patients hospitalized on a medical ward.

### Primary analysis: determinants of carbapenem resistance

We performed the primary analysis on the complete 73 cases and 71 controls. The primary analysis is based on those 144 subjects for whom all potential predictors (listed in Table 1) were completely observed. This restriction is necessary because we compared estimated models to identify the most relevant predictors using the AIC here. All the variables for the primary analysis were of general character (age, sex, comorbidity, etc.) or referred to the time frame of the clinical management prior to when the isolates were sampled (treatment with antibiotics, surgical interventions, etc).

**Table 1:** Complete cases with standardized mean differences (SMDs)

	Sensitive (control)	Resistant (case)	SMD
*n*	71	73	
Carbapenems treatment (days) (mean (SD))	0.8 (2.7)	4.9 (9.2)	0.599
Hospitalization before RT (days) (mean (SD))	12.2 (11.2)	17.9 (14.5)	0.447
ICU before RT (days) (mean (SD))	2.3 (4.4)	5.9 (10.6)	0.450
Surgery, surgery before diagnosis (%)	36 (50.7)	40.0 (54.8)	0.082
Charlson comorbidity index (mean (SD))	2.6 (2.2)	2.5 (2.1)	0.040
Age (mean (SD))	68.8 (15.0)	66.9 (15.3)	0.128
Gender, female (%)	25 (35.2)	29.0 (39.7)	0.093
Previous ICU stay, ICU < 3 m before RT (%)	10 (14.1)	5.0 (6.8)	0.238
Previous hospital stay, hospital < 1 m before RT (%)	27 (38.0)	23.0 (31.5)	0.137
Antibiotics, antibiotics < 1 m before RT (%)	63 (88.7)	67.0 (91.8)	0.103
Amoxicillin-clavulanate treatment (days) (mean (SD))	3.1 (5.7)	2.4 (7.7)	0.111
Piperacillin-tazobactam treatment (days) (mean (SD))	3.3 (5.5)	2.7 (5.5)	0.101
Cephalosporins treatment (days) (mean (SD))	0.7 (1.8)	1.3 (2.9)	0.251
Quinolones treatment (days) (mean (SD))	0.9 (3.9)	1.6 (4.6)	0.174
Macrolides treatment (days) (mean (SD))	0.3 (1.2)	0.2 (0.9)	0.071
Aspirin, aspirin at admission (%)	20 (28.2)	12.0 (16.4)	0.285
Antacids, antacids at admission (%)	27 (38.0)	24.0 (32.9)	0.108
Foreign body (%)			0.439
None	19 (26.8)	27.0 (37.0)	
Arterial catheter	16 (22.5)	21.0 (28.8)	
Prosthesis	3 (4.2)	3.0 (4.1)	
Urogenital foreign body	26 (36.6)	13.0 (17.8)	
Other foreign body	7 (9.9)	9.0 (12.3)	
Transplant, organ/stem cell before RT (%)	3 (4.2)	6.0 (8.2)	0.166
Other antibiotics treatment (days) (mean (SD))	1.3 (3.6)	3.6 (7.1)	0.409
Other antibiotics, intermediate/resistant (%)	5 (7.0)	24.0 (32.9)	0.683

All variables refer to the period before the isolates were taken

*RT* resistance test

Table 1 provides an overview of the analyzed data set. Even though the matching did not explicitly account for age, gender, and comorbidities, the matched data set seems to be well-balanced regarding the variables age (SMD 0.128), gender (SMD 0.093), and Charlson comorbidity index (SMD 0.040). The standardized mean differences of the variables between the group of patients with carbapenem-resistant bacteria and the group with carbapenem-sensitive bacteria are low.

Next, we performed an automated variable selection to identify further relevant variables associated with resistance beside the five pre-selected by the investigators (1st to 5th variable in Table 2). Table 2 shows the odds ratios estimated in the model selected by a forward search. Four additional predictors were selected into the model, which has an improved AIC of 75.61 compared to 87.15 without additional predictors. The duration of carbapenems treatment prior to detecting the resistance appears to be a relevant exposure with an estimated odds ratio of 1.15. This implies that with each day of carbapenems treatment the chances of a resistance to these antibiotics increase by this factor. In addition, we found that resistance to other antibiotics is a strong association (OR 8.33, CI [1.73, 40.13], *p*-value 0.008), while acetylsalicylic acid seems to be associated with a reduction of resistance to carbapenems. Further, the treatment with cephalosporins before the resistance appears to be linked to a higher risk of carbapenem resistance with an Odds ratio of 1.28.

**Table 2:** Odds ratios from conditional logit model considering automatically selected predictors of a resistance to carbapenems with approximate 95% confidence intervals

	Odds ratio	95% CI	*p*-value
Carbapenems treatment (days)	1.15	[1.01, 1.31]	0.036
Hospitalization before RT (days)	1.00	[0.95, 1.05]	0.890
ICU before RT (days)	1.06	[0.95, 1.17]	0.292
Surgery-surgery before diagnosis	1.16	[0.18, 7.43]	0.878
Charlson comorbidity index	0.88	[0.72, 1.08]	0.219
Other antibiotics-intermediate/resistant	8.33	[1.73, 40.13]	0.008
Acetylsalicylic acid-at admission	0.36	[0.10, 1.24]	0.105
Cephalosporins treatment (days)	1.28	[0.96, 1.71]	0.090
Transplant-organ/stem cell before RT	6.44	[0.56, 74.28]	0.136

The predictors were selected based on a forward stepwise model selection, starting from the model with essential predictors (1st to 5th variable)

*RT* resistance test

The performed susceptibility analysis yielded similar results.

### Secondary analysis: clinical implications

The secondary objective was to explore the implications of an infection with carbapenem-resistant, carbapenemase-negative bacteria with respect to a number of clinical outcomes. The descriptive analysis of the secondary endpoints contained the initial dataset of 75 cases and 75 controls. Further secondary analyses are based on 128 to 149 subjects, depending on whether the corresponding endpoints and predictors were completely observed.

Table 3 shows the results of the descriptive analysis of the secondary endpoints. Twelve patients from the sensitive cohort died within 30 days after the antibiotic susceptibility testing had been conducted, and seven deaths of resistant cases were reported. The duration of the whole hospitalization stays and in the ICU was longer in the resistant group. With a day average of 8.6 for cases and 4.5 for controls, the total duration of ICU stays almost half.

**Table 3:** Descriptive analysis of secondary endpoints with standardized mean differences (SMDs)

	Sensitive (control)	Resistant (case)	SMD
*n*	75	75	
Death hospital, died in hospital < 30 d after resistance test (%)	12 (16.0)	7 (9.3)	0.201
Death (%)			0.232
Alive 30 d after resistance testing	53 (70.7)	54 (72.0)	
Died < 30 d after resistance testing	12 (16.0)	7 (9.3)	
NA^2^	10 (13.3)	14 (18.7)	
ICU total (days) (mean (SD))	4.5 (7.9)	8.6 (16.4)	0.315
Hospitalization total (days) (mean (SD))	25.8 (21.2)	34.1 (41.3)	0.253
Re-hospitalization 1 (%)	12 (16.0)	9 (12.0)	0.115
Antibiotics total (days) (mean (SD))	34.9 (42.0)	45.3 (61.2)	0.197

^1^Rehospitalization at the USB within 30 days after the end of stationary care

^2^End of hospitalization within 30 days after resistance testing. No information could be gathered about mortality

Regression models containing the resistance indicator as explanatory factor and controlling for further potential confounders were used to analyze mortality rates in detail. Controlling was conducted with a minimal and an extensive set of confounders. Based on the performance on AIC, one approach was selected and presented in Tables 4 and 5.

**Table 4:** Odds ratios from logit model for death in hospital controlling for a minimal set of confounders with approximate 95% confidence intervals

	Odds ratio	95% CI
Carbapenems-resistant (case)	0.44	[0.14, 1.36]
Hospitalization total (days)	0.96	[0.93, 0.99]
ICU total (days)	1.10	[1.05, 1.17]
Charlson comorbidity index	1.21	[0.95, 1.52]
Age	1.03	[0.99, 1.08]

**Table 5:** Estimated multiplicative effects on ICU stay duration (among those discharged alive) controlling for an extensive set of confounders with approximate 95% confidence intervals

	Estimate	95% CI
Carbapenems-resistant (case)	1.59	[0.81, 3.14]
Charlson comorbidity index	0.94	[0.80, 1.12]
Age	1.00	[0.98, 1.03]
Previous ICU stay-ICU < 3 m before resistance test	1.14	[0.40, 3.22]
Penicillin-BL inhibitor total (days)	1.04	[1.00, 1.07]
Quinolones total (days)^1^	0.97	[0.94, 0.99]
Carbapenems total (days)^1^	1.04	[1.01, 1.06]
Aminoglycosides total (days)^1^	1.01	[0.95, 1.06]
Surgery, surgery before resistance test	0.98	[0.53, 1.80]

^1^Total intake of specific antibiotic subgroups was counted within 30 days prior to resistant test till end of stationary care

With an odds ratio of 0.44 (CI [0.14, 1.36]), the models did not show an association of carbapenem resistance and mortality. As shown in Table 4, the time spent in an ICU during the hospitalization appears to be the most important characteristic associated with dying in hospital.

The association between the length of a potential ICU stay and suffering from an infection with carbapenem-resistant bacteria was assessed using a hurdle model. The model indicates that patients with carbapenem-resistant bacteria tend to stay 1.59 times (CI [0.81, 3.14]) longer in an ICU, when admitted. Potential confounders were included, and the corresponding results are listed in Table 5.

Separate analysis was performed with no remarkable result on duration of total hospitalization stay, rehospitalization at USB, and antibiotic treatment. Adjusted linear regression models did not indicate a difference between patients with carbapenem-resistant bacteria and patients with a carbapenem-sensitive infection.

## Discussion

Most of the previous published literature focused on resistances due to carbapenemases, but very little data exist on the determinants and implications of other possible resistance mechanisms such as porin loss or efflux pump activity in a wider range of Gram-negative bacteria. Therefore, carbapenemase-positive isolates were excluded in this study. The objective of this study was to identify the clinical determinants of carbapenemase-negative, but carbapenem-resistant *Enterobacterales*, as well as *P. aeruginosa*, and to explore the clinical implications of these resistances in a low-endemic setting of carbapenemases. Our analysis shows that the duration of prior carbapenem treatment correlates with resistance to carbapenem. Furthermore, the data indicates that patients with carbapenem-resistant bacteria tend to stay longer in an ICU ward.

With 43 patients suffering from an infection of carbapenem-resistant, carbapenemase-negative *P. aeruginosa*, more than half of the included cases were caused by this pathogen. This is not surprising, considering that *P. aeruginosa* is one of the major nosocomial pathogens and has a well-known resistance mechanism due to porin loss (Sanbongi et al. 2009; Yoneyama and Nakae 1993).

Differences concerning comorbidities between the resistant and the sensible cohort could not be shown. A possible explanation might be that the quantification using the Charlson comorbidity index is lacking in completeness and specificity for infection-relevant comorbidities. For instance, several cases with cystic fibrosis could not be taken into full account due to the index being focused more on chronic respiratory diseases like COPD that are common in the average population. As we are dealing with a carbapenem-resistant subgroup with stated higher prevalence for cystic fibrosis, this could confound the index values (Oliver et al. 2000).

Our first question sought to identify determinants of carbapenem resistance in carbapenemase-negative Gram-negative bacteria. Our models indicate nine predictors with variable evidence. In general, it seems that the development of resistances is the result of numerous interfering risk factors. The analysis of these nine predictors implies that the duration of carbapenem treatment is an important risk factor. Another result is the robust correlation of resistance to carbapenems with resistances to other antibiotics (summing up resistances to aminoglycosides and fluoroquinolones). The multi-resistant bacteria as well as the gradual escalation resulting in the use of carbapenems as antibiotics of last resort explain this temporal association. A strong relationship between the intake of different antibiotics and resistance to carbapenems has been reported in various studies (Adams et al. 2020; Jeon et al. 2008; Schwaber et al. 2008). Interestingly, together with carbapenems, the intake of cephalosporins seems to be associated with a resistance to carbapenems. Kwak et al. stated this association before among carbapenem-resistant *Klebsiella pneumoniae* (Kwak et al. 2005)*.* The exact molecular mechanism was not explored in that study and neither in ours. The increased intake of cephalosporins may correlate with the overexpression of extended-spectrum beta-lactamase (ESBL) or AmpC genes what potentially lead on its own to the resistance of carbapenems or triggered an escalation towards carbapenem treatment (Majewski et al. 2016; Rizi et al. 2023; Wilson and Török 2018). Furthermore, our data suggest the association between the duration of hospitalization in the ICU and the development of resistant species. This seems to be consistent with other research (Schwaber et al. 2008).

We found that the intake of aspirin may be associated with decreased risk of resistance to carbapenems. In discordance with this suggestion, previous investigations (Bandara et al. 2016; Ochs et al. 1999; Zimmermann and Curtis 2018) have indicated that the intake of acetylsalicylic acid is leading to more resistances. Our data could not corroborate the findings of an in vitro study that showed the reduction of OprD porin in *P. aeruginosa* outer membranes and increase of resistance to carbapenems due to acetyl salicylate (Ochs et al. 1999). In addition, another paper investigated the outer membrane proteomic profile of a *P. aeruginosa* isolate and found that some porins were downregulated in presence of salicylic acid in vitro (Bandara et al. 2016). It remains unclear if this in vitro observation is clinically meaningful. In our study, it could be that the intake of acetylsalicylic acid is associated indirectly with a lower exposure to carbapenem antibiotics. Further studies are needed to explore the potential impact of co-medication on antibiotic resistance.

Next, we examined the clinical impact of carbapenem resistance. The analyzed dataset does not provide evidence for strong clinical implications of a resistance to carbapenems. The results of the secondary analysis propose that patients suffering from infection with carbapenem-resistant bacteria have longer ICU stays. As a stay in the ICU increases hospitalization costs dramatically, this may confirm the association of carbapenem resistance and higher hospitalization costs in earlier research (Adams et al. 2020). The results of the descriptive analysis regarding the hospital stay duration could not be confirmed with models considering multiplicative effects. In this case, we could not support evidence from previous observations (Adams et al. 2020).

Evidence for an increased mortality could not be demonstrated. This may be explained by a previous study which suggests poorer outcomes for carbapenem resistance due to carbapenemases compared with resistance of non-carbapenemase-producing *Enterobacterales* (Tamma et al. 2017).

However, these results need to be interpreted with caution. Our study has the following limitations: retrospective data with absence of further follow up after the stay in the hospital of tertiary care and limited number of patients. The limited number of patients from a single center in Switzerland and the exclusion of carbapenemase-positive cases may lead to reduced generalizability, especially in other endemic settings (European Centre for Disease Prevention and Control 2018; Federal Office of Public Health and Federal Food Safety and Veterinary Office 2018). We cannot rule out the possibility of confounding due to unmeasured variables, e.g., other interventions like ERCP. Also, we could not perform detailed molecular analysis such as transcriptomics to fully understand the reason for the carbapenem resistance, e.g., overexpression of ESBL or AmpC genes. Nevertheless, we focus in detail on an important group of microbiologically and clinically relevant bacterial strains with carbapenem resistance in absence of carbapenemases.

In conclusion, the duration of prior carbapenem treatment seems to be a strong risk factor. Furthermore, the higher risk for a longer ICU stay could be a consequence of a carbapenem resistance. Considerably more work, especially with larger cohorts, will be needed to further corroborate risk factors for the development of resistances of non-carbapenemase-producing gram-negative bacteria as well as their clinical implications.

### Supplementary information

ESM 1

## Data Availability

The datasets used and/or analyzed during the current study are available from the corresponding author on reasonable request.
