# Supplementary material for: Factors associated with non-carbapenemase mediated carbapenem resistance of Gram-negative bacteria: a retrospective case-control study
Source: Int Microbiol. 2023 Aug 9;27(2):597–606. doi: 10.1007/s10123-023-00405-6 (PMC10991015; doi:10.1007/s10123-023-00405-6)
Supplement: Supplementary file 1 — ESM 1 [file 10123_2023_405_MOESM1_ESM.docx]

| **Bacterial species** | cases/controls | **Ward** | cases/controls | **Isolation site** | cases/controls |
| --- | --- | --- | --- | --- | --- |
| n | 75 | n | 75 | n | 75 |
| *Pseudomonas aeruginosa* | 43 | Medical ward | 30 | respiratory material | 31 |
| *Enterobacter* spp. | 22 | ICU | 23 | urine | 19 |
| *Klebsiella* spp. | 4 | Surgical ward | 22 | deep tissue | 17 |
| *Serratia* spp. | 3 |  |  | superficial swabs | 6 |
| *Escherichia coli* | 2 |  |  | blood | 2 |
| Other Enterobacteriaceae^1^ | 1 |  |  |  |  |

***Table S1.*** *Numbers of cases/controls according to the exactly matched criteria. Deep tissue, primary sterile sites including e.g., biopsies, cerebrospinal fluid, etc; superficial swabs, including swabs from wounds, etc.*

^1^ *Citrobacter freundii*
